# A review on Respiratory allergy caused by insects

**DOI:** 10.6026/97320630014540

**Published:** 2018-12-22

**Authors:** Mohd Adnan Kausar

**Affiliations:** 1 Department of Biochemistry, College of Medicine, University of Hail, Hail, Saudi Arabia, KSA

**Keywords:** Allergens, respiratory allergy, insect allergens, Mosquito allergens, ockroach allergens

## Abstract

Hypersensitivity or allergy encompasses a wide range of immunological reactions that generally have adverse consequences involving one or many organ systems of the body. Allergens are usually glycoprotein or chemically complex low molecular weight substances. The common allergens include pollen, fungal spores, house dust mite and house dust, animal danders, drugs, foods, insect emanations, and detritus, etc. Information on the role of insects in respiratory allergy is increasing in the literature. There are about 30 million living species of insects. These insects can broadly be classified as stinging insects, biting insects and non-stinging and non-biting insects. All materials form insets namely wings, scales, saliva; dried feces and venom can cause allergic diseases, such as rhinitis, conjunctivitis, asthma and urticaria. There are wide varieties of insects such as moths, butterflies, bees, wasps, hornets, yellow jackets, flies, beetles, cockroaches, and mosquitoes. Exposure to emanations and detritus of these insects may lead to several allergies in some genetically predisposed individuals. Therefore, it is of interest to review allergies caused by various insect’s stings and bites and their adverse effect on the human body.

## BacAllergy and Insect:

Allergens are usually proteins or glycoprotein or chemically complex substances with low molecular weight. Their molecular complexity, concentration, solubility and stability in body fluids were other important determinants of allergenic potential 1. The common allergens include pollen, fungal spores, house dust mite, and house dust, animal dander, insect emanations and detritus, drugs, foods, etc. Out of these, the allergenic significance of a large number of pollen grains, fungal spores, animal danders, house dust and house dust mite has been extensively studied all over the world including India and was very well established 2-9. The role of insects as sources of inhalant allergens insects was also well studied and suggested insects were one of the most important sources of aeroallergens.Insects, an important class of the phylum Arthropoda, were characterized by an exoskeleton, a body showing segmentation and bilateral symmetry and jointed appendages. The numbers of insects' species were more as compared to any other group of animals. An insect mainly highlights the world most diverse group and numerous classes of the animal kingdom and includes a number of species i.e. praying mantis, dragonflies, grasshoppers, true bugs, flies, fleas, bees, wasps, ants, lice, butterflies, moths, and beetles. The number of species of insects was estimated to be between 6 to 10 million with more than a million species already discovered. They assume the role among more than half of all living organisms that were known presently and potentially serve as more than 90% of the different forms of life on Earth. Hence, contacts of the human with insects were inescapable. Human exposure to biting or stinging insects or to their remains may range from conditions in which they were barely noticeable to severe lifethreatening conditions. From studies conducted by Terry Erwin of the Smithsonian Institution's Department of Entomology in Latin American Forest Canopies, the number of living species of insects has been estimated to be around 30 million 10. Insects fall under 33 orders, which were further divided, into 839 families 11. Of all the insects, moths, butterflies, bees, wasps, hornets, yellow jackets, flies and mosquitoes constitute about 40 percent, the beetles another 40 percent and the rest about 20 percent 12. These insects can be broadly be classified as stinging insects, biting insects and non-stinging and non-biting insects 13. All insect matter like wings, scales, saliva, dried fecal matter, and venom can cause allergic diseases such as rhinitis, conjunctivitis, asthma, urticaria and gastric disorders 14,15 . Depending on the route of sensitization, the insect allergens have been recognized as an inhalant, ingestant and injectant allergens 16.

Insect’s leads to a number of allergies that in turn results in pain, itching and appearance of redness and swelling at the bite/sting or surrounding affected areas. It has been reported that people allergic to stinging venom may possess certain serious reaction namely anaphylaxis 17,18 . It has been reported that from the last decade the number of patients with respect to insect allergy has increased. However, mortalities have been known to reduce mainly due to improved diagnosis and upgraded treatment procedures. The socio-economic burdens linked with insect-related allergies were still unknown. Insect prone allergies were also known as Hymenoptera Venom Allergy (HVA). The HVA allergies have been known to cause large local reaction (LLR) or systemic allergic responses. The induced allergic responses affect the local area and result in the depth of more than 10cm within 24 hours at the sting site. Hymenoptera mainly belongs to the sub-order Aculeate and constitutes several super-families namely Apoidea, Vespidae, and Formicidae [18]. The common insect’s varieties in these families include: (1) Yellow Jackets; (2) Honeybees; (3) Paper wasps; (4) Hornets. The allergens which mainly initiates allergic response via honeybee sting were phospholipase A2 (Api m 1) and hyaluronidase (Api m 2). Allergens in yellow jacket’s venom include (1) Phospholipase A1 (Ves v 1); (2) Hyaluronidase (Ves v 2); (3) Antigen 5 (Ves v 5). Allergens particularly found in Fire ants include: (1) Sol r 2 (A Phospholipases); (2) Sol i 2; (3) Sol i 3; (4) Sol gem 2.

## Insect bite and sting:

Insect breaks or punctures in the skin via bite and/or sting. The conditions became complicated when insect introduce their saliva, venom or excretory products into the skin through puncture. The specific components present in these injected substances were prone to give rise to an allergic reaction. These allergic reactions, in turn, result in the appearance of skin lesions that may vary from a typical small itching wheal or slightly elevated area of the skin to further large painful areas of inflamed skin covered thoroughly by vesicles and crusted lesions. The member of flying insects namely flies, gnats and mosquitoes mainly attack the exposed parts of the body. Each bite results in a single itchy wheal that subsequently diminishes within hours. Crawling insects may attack any part of the body including the covered areas of the body and generates characteristic skin diseases particular to each insect variant. Scabies or sarcoptic itch invades the skin and leads to inflammation particularly by the itch mite, Sarcoptes scabiei. The female mites attack the skin and burrows beneath the superficial layer of the skin to lay its eggs in a tunnel visible as a dark wavy line. This lesion initially becomes intensely itchy. After a couple of days to few months, the scratching further develops into secondary skin lesions with papules (solid elevations), pustules and crusted skin areas. The itchiness that appears was caused due to the accumulation of fecal deposits by the mite in the burrow region. Scabies is commonly observed between the fingers, other persistent locations besides fingers being the natural folds of the skin and pressure areas.

The symptoms of a severe systemic allergic reaction to an insect sting include: (1) A sudden feeling of weakness (caused by a drop in blood pressure); (2) Dizziness; (3) A sense that something terrible is happening; (4) A rapid pulse; (5) Swelling of the airways and throat, making it difficult to breathe; (6) Severe asthma; (7) Itching and swelling away from the site of the sting; (8) Stomach cramps and/or a feeling of sickness Pediculosis is the skin disorder caused bloodsucking lice. These bloodsucking lice belong to various species and infect the scalp, groin, and body. The lice invade near or onto the skin and attach their eggs to the hair or clothing of the host on which they frequently feed. As a result, a small itchy red spot appears and may further become infected after repeated scratching. Chiggers i.e. the larvae of certain mites’ were an inhabitant on humans and suck the blood. The bite of chiggers produces a wheal on the skin with intense itchiness. The itchiness has been known to occur as a result of the digestive juices of the chiggers being injected while feeding blood. Other bloodsucking insects that in habitat humans were fleas, bedbugs and ticks, that originally lives in the ground, bedding, walls, and furniture and temporarily act on humans as primary hosts. The most commonly observed lesions on humans were of bedbug and fleas. Bedbug produces a burning wheal sensation with the central punctured dot. The flea results in a cluster of wheals and papules since fleas inject several adjacent spots in the course of feeding on the skin. Insect’s sting generated by the family of stinging insect results painful swelling of the skin and the severity of the lesion varies according to the site of the sting and the type of the insect. A variety of species of bees and wasps belongs to the family of stinging insect. They possess two poison glands. One gland is engaged in secreting toxin with formic acid as one the most recognized constituent. The other gland secretes an alkaline neurotoxin that acts independently. The secreted toxins were individually mild by nature, but when grouped and injected together via stinger, the combination leads to strong irritating properties. In some cases, the bee or wasp sting causes a severe allergic reaction known as anaphylaxis. Other examples of stinging insects were Hornets, some ants, centipedes, scorpions, and spiders. Some insects leave their sting in the wound. In such cases when multiple stings were being injected, it may give rise to severe systemic symptoms and in severe cases may lead to death. The bites of some spiders were reported to be lethal, particularly in young age group children.

## The Global Prevalence of Insect Allergy:

The prevalence of insect allergy worldwide is approx 1 to 7% 19 with more ubiquity reported among the middle and old-age population 20,21. The average pervasiveness was mainly to large local reaction of about 2.4% -24.6% among general population up to 38% in beekeeper’s population. In US, the common inset derived allergies were caused by Paper wasp, Yellow Jacket, Hornet and European Hornet with the prevalence of 0.5- 3.3%. According to world allergy organization, around 40 deaths per year in US were reported to be occurred as a result of insect allergy. The prevalence percentages of insect-borne allergy were reported to be higher in UK as compared to US by 11.5% people being infected 22. The most preferred allergy among the entire key allergy includes beewasp allergy with 2% prevalent in the population. Other than beewasp allergy, hornet imposed allergic reaction was reported in UK population 23. Furthermore, in Australia, nearly 15-25% of the total populations were diagnosed with different insect allergies. The major cause of allergy being from ant stings mainly Australian Jack Jumper Ant 24. In Japan, more than half of the total populations i.e. 61.5% were reported to be affected by insect allergy leading to high fatality rate 25. The increased percentages of insect prevalence were reported in the case of Africa with 28%. Among the common allergies, the most preferred allergic reactions were known to be caused by black flies, which are responsible for the transmission of onchocerciasis 26.

The estimated prevalence of subjects with experienced immediate systemic reactions to insect stings varies from 1-7% of the total population. The insects that was responsible and accountable for the sting related problem was found to vary by region. The honeybee is ubiquitous, yellow jackets (Vespula) were responsible for sting allergy problems across diverse temperate climates, paper wasps (Polistes) and hornets (Vespa) tend to be important at lower latitudes. Stinging ants were more restricted by distribution, with exception of the imported fire ant (Solenopsis invicta) and jack jumper ant (Myrmecia pilosula spp) that dominates clinical sting allergy in areas of South Eastern-South Central USA and South Eastern Australia respectively. The species of Pachycondyla results in major public health problems in East Asia and the Arabian Peninsula region. Recently, anaphylaxis resulting from the bite of the paralysis tick, Ixodes holocyclus has been reported as an emerging problem on the east coast of Australia and has been found to be associated in many subjects with subsequent anaphylaxis to red meat. There have been many reports of anaphylaxis to tick bites in Europe. It has been found that bites resulting from Ixodes in Australia and Europe and Amblyomma and Dermacentor spp in North America were important stimuli with respect to sensitization to the carbohydrate allergen, galactose alpha-1,3 galactose. It has been also reported to be widespread in non- primate animals and also linked to allergic reactions to the monoclonal antibody cetuximab. Anaphylaxis related to biting insects like kissing bug (Triatoma), horseflies (Tabanus spp) and mosquitoes (Aedes, Culex and Anopheles) has been rarely reported. The estimated numbers of deaths resulting from the sting anaphylaxis were likely to vary widely regionally. It has been reported to lie most likely in the range of 1-10 per 10 million per annum based on estimates in the USA and Australia. The prevalence of insect allergy in Indian sub-continent was reported to be 30% i.e. one-third of Indians from the total population was suffering from insect allergies mainly due to huge diversity and lack of basic prevention strategies. The most common insect allergy prevalent in India is mainly due to honeybee stings. The common allergies prevalent across the major countries around the globe were summarized in Table 1.

## Diagnosis and Therapeutic review of insect allergy:

The diagnoses of insect sting were mainly judged through limited swelling of the local area being stringed. However, mostly only in minority of cases, the swelling can prolong by 24-48 hours. Furthermore, only a small percentage of individuals develop systemic reaction beyond the area of sting leading to IgE mediated reaction. Hence, diagnosis in case of insect allergy is crucial for the treatment of individuals. Among the common diagnostic tests being used, skin test is the most common with 90-95% accuracy [17]. However, there is a need of specialist evidence regarding the diagnosis with sting because the availability of venom-specific IgE is difficult. Thus, in such case sensitivity test for different types of insects should be carried out. Consequently, another test that is common besides skin test in the diagnosis of the disease is the measurement of Mast Cell Tryptase. The measurement should be carried out within 30- 38 hours of the sting bite in order to conclude an allergic reaction 27. At last, another diagnostic tool that has been particularly in use but not widely used is sting challenge mostly applicable in clinical practice. In India, blood test and skin test were most commonly used to determine insect-specific allergic reactions.

In mild cases of insect allergy, anti-histamines were given with oral prednisolone. Further, H1 and H2 antihistamines were used to enhance the effect of diphenhydramine 28. However, in case of severe infection, venom immunotherapy was given as a treatment procedure. Epinephrine injections were also considered as management for the allergy 29. Local reaction such as cold compresses, elevation of the affected limb, anti-inflammatory, and oral corticosteroids can also be used 30. In India, Japan and UK, several medicinal plants have been used for the treatment of insect bite. They use the extract from the plant such as terpenoids, flavonoids, tannins, and cardiac-glycosides. These plants have triple effect of anti-bacterial, anti-inflammatory and anti-viral reaction 31.

## Insects sting allergy and Neuro problems:

The exposure of humans to insects or insect material may lead to the allergy that can be natural, domestic, hobby-related and occupational 32. Prior to 1960, a few scattered reports on asthma or rhinitis due to exposure to insect allergens were available. These insects include may fly, aphid, caddisfly, housefly beetles etc 33-36. Feinberg and coworkers (1956) carried out skin tests with insect extracts on a large number of patients suffering from asthma, hay fever, atopic dermatitis, and conjunctivitis and reported that most of them showed a positive response, indicating that the dust of disintegrated insects might be an important cause of inhalant allergy [36]. After that, a number of studies with high incidence of skin positivity to insect extracts were reported among patients of bronchial asthma or allergic rhinitis 37-39.

A number of stinging insect’s allergic reactions in humans have been also reported. The most commonly studied includes wasps namely hornets (Vespa), yellow jackets, European wasps (Vespula) and paper wasp (Polistinae); bees namely honey (Apis mellifera) and bumble bees (Bombus) and stinging ants such as fire ants (Solenopsis), jack jumper and bull ants (Myrmecia, and Pachycondyla) 40. The basic sting reaction comprises of three types and the symptoms were dependent on the site of sting. Stings in the mouth may cause serious airway obstruction even in case of people who were not hypersensitive to the venom. Systemic manifestations may include hypotension, broncho-constriction, respiratory distress, syncope, laryngeal edema and death. The classified were based on the severity of grade of insect sting ( Table 2). The normal sting reaction where the area around the bite becomes red followed by itchiness and severe pain; a large local sting reaction with swelling areas greater than 5 cm and systemic reactions with symptoms arising in areas other than the area of bite 41. In some cases, a large local reaction may occur with skin redness greater than 10 cm 42. It can last for about two days and known to be occurred in about 10% of bitten cases 43. In case of some persons, excessive local swelling may develop that can be either immediate i.e. developing and peaking within 1-2 hours or can be delayed developing after 24 to 48 hours of the sting and eventually resolve after 3-10 days of biting. A small range of the population may develop systemic or generalized reactions extended beyond the neighboring areas. These were mostly immediate IgE-mediated allergic reactions and mainly comprises of cutaneous, mucosal, respiratory, cardiovascular, gastrointestinal and neurological involvement ( Table 3). Systemic reactions have been classified by the four-stage system of Ulrich Mueller with a modification in the system of Harry Mueller described earlier.

Nowadays, Simon Brown described a new three-tier system ( Table 2) describing the significance of features that increase the probability of occurrence of hypoxia or hypotension. Recently, WAO has proposed a classification covering standard reports of reactions to subcutaneous immunotherapy 44. Human exposures to insects were often associated with most notable immediate risk like anaphylactic shock. Hypersensitivity arising as an outcome of harmless insect saliva, venom, body parts, excretions or secretions may result systemic responses in case of some individuals. Diagnosis of the early phases of the systemic allergic reaction leading to an anaphylactic shock is of great importance in terms of treating any patient suspected with insect exposure. Anaphylaxis may be fatal in the time duration of 10 minutes in severe cases. The rate of reoccurrence is around 40-60% in insect stings 45. In India, systematic detailed research on the significance of insects in the etiology of allergic respiratory disorders and their treatment by immunotherapy was initiated in 1969 by Shivpuri and coworkers 46. Shali and coworkers (1970) reported the presence of some sexspecific allergens and antigens in the WBE (Whole Body extract) of male and female cockroaches (Periplaneta americana) 47.

They gave evidence for the presence of sex-specific allergens in the cockroach. In 1971, Shivpuri and coworkers published the results of another detailed study on insect allergy 48. Subsequent extensive research conducted by Agarwal and co-workers in the field insect allergy conclusively established that insects play an important role as sources of inhalant allergens in the etiology of allergic respiratory diseases 49-56. Chaudhry (1988) conducted a systematic and comprehensive study on the clinico-immunologic properties of twelve insects and reported that all these insects play an important role in type I allergic respiratory disorders 57. These insects included cockroaches (Periplaneta americana and Blattella germanica) male and female; housefly (Musca domestica); locust (Schistocerca gregaria) male and female; mosquitoes (Aedes aegypti, Anopheles stephensi and Culex quinquefasciatus) and moths (Heliothis armigera and Spodoptera litura). It has been reported that venoms and saliva of insects play an important role in the etiology of allergic respiratory diseases. Venom hypersensitivity may not only be mediated by immunologic mechanisms (IgE-mediated or non-IgEmediated venom allergy) but also by non-immunologic mechanisms 58. Several major allergens usually glycoprotein have been identified in venoms of bees, vespids and ants 59. The structures and sequences of the majority of venom allergens have been determined and several have been expressed in recombinant form 60,61.

The insects belonging to Hymenoptera order comprises of wasps, bees and ants inject their venom via stinging. The venom injected by distinct species has been found to differ in terms of biochemical and immunological backgrounds, however; cross-reactivity between some species has been found 62. Wasp venom contains thrombogenic, vasoactive and inflammatory peptides; amines; enzymes; low molecular weight compounds i.e. serotonin, histamine, and acetylcholine. The occurrence of reactions as an outcome of venom injection might be local, regional, systemic anaphylactic and delayed-type hypersensitivity 63. The venom of insect’s wasps, bees and ants induces acute IgE-mediated type I or type III hypersensitivity reaction with the impeachment of immune complexes and complement activation. These reactions were mainly delayed and reported to occur within days to several weeks after the inoculation of venom. Neurologic symptoms governing Hymenoptera stings were mainly uncommon, but several cases have been reported globally with both central and peripheral nervous system involvement. These can be cranial neuropathies, acute inflammatory polyradiculoneuropathy, stroke, encephalitis and myasthenia 64. There have been reports about wasp sting– induced allergic encephalitis worldwide with 2 from Russia and 1 from India 65. In these reports headache and seizures and response to steroids were observed 66. Although hypothalamic hamartoma, gelastic seizures associated with different cortical foci i.e. frontal, temporal, and parietal were also reported 67. It has been found that in case of stings incorporating by a large swarm of stinging insects may lead to mass envenomation and the patient must be treated aggressively and observed within 12 to 24 hours for the development of coagulopathy, renal and neurological damage.

## Clinical studies with different insects:

### Moths and Butterflies:

The order Lepidoptera comprises of moths and butterflies and their larvae i.e. caterpillars. There have been an estimated 125,000 to 150,000 different species of moths and butterflies in this order. However, few were capable of inducing adverse reactions in humans. Caterpillars were mainly responsible for the majority of adverse reactions occurring among humans. Adult moths and butterflies do not appear to cause any adverse reactions in humans. The Lepidoptera order possesses two sub-orders i.e. Order Rhopalocera comprises of adult specimens that fly during daytime and were commonly known as butterflies and Order Heterocera with nocturnal activities and were called as moths. In a study comprising lepidopterans (butterflies and moths) derived skin lesions found that lesions were found to be appear by two mechanisms. First, through contact with irritating hairs or setae of some caterpillars and secondly by the action of body setae of adult moths, that were considered rare 68,69. Their phases of development include egg; larva or caterpillar, pupa or chrysalis and an adult phase called imago and represent a complete or holometabolic evolution 70,71 . Generally, the pathophysiological complications related to toxic moths and caterpillar exposures were classified into several clinical syndromes. These include erucism i.e. disorder occurs as a result of reactions due to caterpillars; lepidopterism comprises of cutaneous and systemic signs; pararamose with severe arthralgia and arthritis due to spp. of Premolis caterpillars; lonomism due to contact with Lonomia caterpillers; dendrolomiasis due to exposure to Dendrolimus caterpillars and ophthalmia nodosa with ocular involvement 72-74 (Table 4). The caterpillar-induced bleeding syndrome is a unique disorder belonging to the Lonomia genus, a type of moth native to South America. Around 688 cases of caterpillar envenomation were reported in the state of Rio Grande do Sul in Brazil during periods of 5 years 75. These bleeding syndromes were known to cause by two species belonging to Lonomia caterpillars 76. They were L. oblique, native to southern Brazil and L. achelous commonly found in Venezuela and northern Brazil. Both caterpillars were reported to induce a consumptive coagulopathy and bleeding syndrome in a similar way. Although the pathophysiologic processes behind the occurrence of the bleeding syndrome were not fully known, the mechanism via which this occurs was reported to be slightly dependent on the causal species. Caterpillar-induced bleeding syndromes were characterized by initial symptom like mild fever, local burning pain, headache, nausea, and vomiting 77. As clotting factors were consumed via venom-induced activation of the coagulation system, within 1 hour to around 10 days several bleeding complications arise. These bleeding manifestations include mucosal hemorrhages, hematuria, and ecchymosis after envenomation. In case of Lonomia envenomation alveolar hemorrhage, acute renal failure and intracranial hemorrhage occur 78.

When poisoning was linked with caterpillars of the species L. obliqua, the signs and symptoms after bristles contact includes severe pain with burning sensation, redness and swelling at the site of contact site, general malaise, vomiting, shortness of breath, bruising, mucous membranes bleeding, epistaxis, melena, hematuria, anuria, hypotension, headache, arthralgia, myalgia, back pain, weakness and fever 79,80. A number of cases were reported with allergy to these two insects 81.

Parlato (1932) was the first to report respiratory allergy caused by moths and butterflies 82. He reported that scales and hair of these insects (Lepidoptera) were commonly found in the air. Studies conducted by Kino and Oshima also implicated these insects as the possible cause of respiratory allergy 83-85. They found that more than half of the randomly selected asthmatic population showed not only positive skin tests and RAST but also positive bronchial provocation reactions to these outdoor insects. They did not find any cross-reactivity between house dust mite and moth allergens. Wynn and coworkers (1988) immunochemically measured the air born concentration of Lepidoptera allergen and concluded that moth might be a seasonal allergen 86. Araujo and coworker (2014) reported high frequency of sensitization to Bombyx mori (Moth) in a selected population of patients with respiratory allergic diseases 87. Wills and co-workers (2016) reported that allergic diseases are a disease caused by the scales and toxic fluids of adult moths and butterflies 88.

### Midges

Midges were tiny flying insects with 2-3 mm length wingspan. Around the world, 20,000 species of midges were identified and the majority of them were non-biting midges. A female midge has been only known to bite, as they need blood to feed their eggs. Male midges only invade plants and suck plant nectar. Culicoides is a genus of biting midges belonging to the ceratopogonidae family. There were over 1000 species in this genus 89 divided into many sub-genera. Several species were known to act as vectors of various diseases and parasites eventually affecting animals. Culicoides biting midges (Diptera: Ceratopogonidae) are among the smallest blood-sucking flies 90. The Culicoides biting midges in public health were reported to biologically transmit Oropouche virus (OROV), the etiological agent of the febrile illness Oropouche fever among human beings 91. The most commonly observed symptoms of Oropouche fever were mainly headache in most of the cases, followed by generalized arthralgia, anorexia and in severe cases meningitis, the incidence of which were presently unknown in the majority of epidemics 92. The distribution and incidence of endemic OROV were currently under review and were found to be somewhat linked to the recent discovery in Peru of Iquitos virus, which possesses similar clinical manifestations but the mode of transmission has been yet to be investigated in detail 93. In addition to OROV, Culicoides also play an important but limited and poorly defined role in the transmission of many other zoonotic arboviruses, which were of paramount importance. Other human pathogenic arboviruses have also been detected in fieldcaught adult female Culicoides and oral susceptibility has been found for Rift Valley fever virus (RVFV), following initial detection in field populations.

The two cases of Chironomus thummi allergy were reported by Baur and co-workers (1980) 94. They reported that 20% of sera from 642 chironomids exposed subjects developed immediate-type hypersensitivity and had significantly raised levels of specific IgE antibodies. Later several other reports were published from various parts of the world suggesting that chironomid particles were one of the important inhalant allergens causing asthma 95. Contact with these insects takes place where there are abundant water i.e. Japanese rice fields 96, hill region of Sudan 97 and lake areas in Wisconsin, U.S.A. 98. In Germany, Chi-t 1-9 allergy was widespread among fish breeders because freeze-dried chironomid larvae were frequently used as fish food. Similar, observations were made by Baur and Liebers (1992) who observed that people handling fish food frequently suffer from asthma 99. Several other reports indicated that chironomid bodies and scales get disintegrated into small pieces and become airborne causing allergic respiratory diseases 100. Other reports demonstrated that adult, as well as larva of these insects may cause respiratory diseases in sensitized persons and larvas were found to be more allergenic as compared to the adults 101. Haemoglobin of chironomidae has been found to induce IgE-mediated diseases 102. Nandi and coworker (2014) reported that the chironomid midges Chironomus circumdatus and Polypedilum nubifer can elicit sensitization in humans 103. The ability of the midge to induce allergic symptoms in intensely exposed population was investigated in Sudan. Where the prevalence of rhinitis and asthma was found to be higher in green nimitti midge (Cladotanytarsus lewisi) exposed villagers 104. A similar midge species, Chironomous plumosus was found to be responsible for respiratory allergy in 45 percent of heavily exposed atopic patients. Both adult and larval organisms were shown to contain the major allergen 105. In Japan, studies confirmed that 38 percent of 303 asthmatics in Tokyo and 58 percent asthmatic children in Toyama showed skin reactivity to different species of midge. Midge specific IgE antibodies were found in 32.4 percent and 41.9 percent of the total patients 106. Using various components of hemoglobin of midge larvae including purified Chi t I, studies were undertaken to identify T cell epitopes involved in allergen-specific stimulation of human peripheral blood lymphocytes. Lymphocytes of each patient showed an individual stimulation pattern probably due to genetic restriction 107.

### Mosquito Allergy:

The mosquito name was derived from a Spanish word, which means “small fly.” It belongs to the family Culicidae. There were thousands of species of mosquitoes known so far, with females possessing the distinguishing characteristic of having a tube-like mouthpart known as a proboscis. This proboscis pierces the skin of the host to draw blood. Female mosquitoes require the nutrients mainly vitamins in blood to produce eggs. Mosquito allergies were highlighted by intense local skin symptoms including not only erythema or bulla but also ulcer or scar with general symptoms of high fever followed by mosquito bites. Most of the cases of mosquito allergy were reported from East Asia and the majority of patients were found to be dying of hemo-phagocytic syndrome 108. The reaction to mosquito bites arises as a result of an immunologic response to proteins present in mosquito saliva. Many people who were known to be bitten by mosquitoes develop an immune response for these proteins; however, only a small proportion of them develop clinically relevant allergic reactions, in common large local reactions 109. There are two main types of reaction arises as a result of mosquito bites. First is the Typical (normal) reactions in which local cutaneous reactions occurs consisting of immediate wheals or swelling with surrounding flares (redness) peaking at 20 minutes; delayed itchy and indurated (firm) papules peaking at 24 to 36 hours and eventually diminish over 7 to 10 days. Second is the large local reaction to mosquito bites. Large local reactions were far most common type of allergic reactions to mosquito bites. These were also termed as Skeeter Syndrome; typically consisting of an itchy or even painful area of redness, warmth, swelling, and indurations that ranges from a few cm to more than 10cm in diameter. Large local reactions develop within hours of the bite with subsequent progress by 8 to 12 hours or more and resolve within 3 to 10 days. Large local reactions may cover the peri-orbital region and much of the face or even entire face, especially in case of an infant or child. They can further interfere with seeing, eating, drinking or normal use of extremities. Severe large local reactions can be represented by low-grade fever and malaise. Systemic allergic reactions to mosquito bites include papular or acute generalized urticaria. In rare cases, severe asthma, anaphylaxis, serum sickness or lymphadenopathy, hepatosplenomegaly, fevers and necrotic skin reactions at the site of mosquito bite may be seen 110.

Mosquito bites can cause varying levels of local swelling, papular urticaria in case of children and rare systemic allergic reactions reaction most often seen in children arises as a result of mosquito and flea bites. Although, a variety of other bites have been also reported to be linked in smaller numbers with the occurrence of hypersensitivity reactions. Systemic allergic reactions may arise in response to the mosquitos’ bites, several types of bloodsucking flies, fleas, kissing bugs, lice and ticks 111. Sometimes, the bites of these insects lead to serious health problems like paralysis, malaria, encephalitis and West Nile Virus and others. These bites may also cause life-threatening and traumatic conditions, if insect allergic persist itself or if the causal organisms invade inside the body upon biting the skin. Malaria, dengue, West Nile virus, chikungunya, yellow fever, filariasis, tularemia, dirofilariasis, Japanese encephalitis, Saint Louis encephalitis, Western equine encephalitis, Eastern equine encephalitis, Venezuelan equine encephalitis, Ross River fever and Barmah Forest fever were group of known disorders arises as a result of mosquitoes sting. People with neuro invasive West Nile virus can develop conditions such as encephalitis i.e. inflammation of the brain or meningitis i.e. inflammation of the surrounding tissue of the brain. The main symptoms include headaches, fever, neck stiffness, disorientation, coma, tremors, seizures and even paralysis.

A number of allergic reactions to mosquito bites have also been reported by various researchers 112,113. Allergic reactions to mosquito bites follow the classical pattern for allergic disease. Predisposed individuals with no previous exposure to mosquito give no reaction to an initial mosquito bite but they do soon subsequent bites 114. The reactions were usually Type I, immediate hypersensitivity responses, but may also induce delayed local cutaneous responses 115 and even anaphylaxis. Immediate and/or delayed reactions to mosquitoes were induced by components of the salivary secretions. Kausar and coworker (2007) reported mosquito as sources of inhalant allergens 116. They also reported the clinic-immunologic and immunochemical characterization of mosquito Whole Body Extract (WBE). There were very few reports about mosquito body parts (such as scales, wings, particulate fragmented body parts) getting aerosolized and inhaled resulting in sensitization by genetically predisposed individuals and causation of allergic diseases 117. Mosquitospecific IgE in the sera of patients and IgE binding allergenic components in the mosquito WBE (Aedes, Anopheles and Culex species) has been reported by Agarwal 1991 and Wu and Lan 1989 117. Kausar and coworkers (2007) performed immunoblot analysis with WBE of 3 species of mosquitoes (Culex quinquefasciatus, Aedes aegypti and Anopheles stephensi) and found unique individual IgE-binding patterns and suggested that both genus and species-specific mosquito allergens exist [116].

### Wasp and Bee Stings

Hymenoptera stings were the common cause of severe allergic reactions ranging from local reactions to anaphylactic shock or even death in some cases 118. Wasps belong to the order of Hymenoptera and include ants, apids (bees and bumble bees) and vespids (wasps, hornets and yellow jackets). Allergic reactions to Hymenoptera stings range from several local to severe systemic reactions or even death. These reactions were usually acute, beginning within minutes to hours reported in around 76–96% of the patients. However, there were reports of delayed responses occurring after several days to weeks of the event. Of the 2606 reactions noted in 1964 by Academy of Allergy survey, 2.8% did not appear until several days after the sting. There have also been reports of neurological complications, hyperglobulinaemia, thrombocytopenic purpura, nephrotic syndrome and hepatorenal syndrome. The neurological complications were infrequent but often serious and include clinical manifestations damaging the central and peripheral nervous systems. Means et al. reported a case with relapsing and progressive course of neurological symptoms and signs including bilateral weakness and numbness of the arms and legs followed by sting by yellow jacket (Vespula pennsylvanica) 119. The patient was found alert and oriented throughout the clinical course, but eventually died after sudden respiratory and cardiac arrest. Necropsy revealed massive pulmonary embolism as the cause of death. Examination of the nervous system showed areas of demyelination throughout the central and peripheral nervous system associated with necrosis and inflammatory infiltrates in the brain stem and spinal cord. Maltzman et al reported about two cases and reviewed other five cases of optic neuropathy after the sting by bee and wasp 120. Most cases reports significant visual recovery after corticosteroid treatment. Bachman et al. reported five cases with acute inflammatory polyradiculopathy following Hymenoptera stings with good recovery 121. Some cases had nerve biopsy, which showed segmental demyelination. Several serious neurological manifestations and cerebral lesions of Hymenoptera stings have been also reported. Encephalitis 122, peripheral neuritis 123, optic neuropathy 124, myasthenia gravis 125, cerebral infraction 126, acute inflammatory polyradiculopathy indistinguishable from Guillan-Barre 127, acute disseminated encephalomyelitis 128 and encephalo-myelo-radiculoneuritis 129 all have been reported. Means et al. 130 reported a case with relapsing and progressive bilateral weakness and numbness of arms and legs followed by Vespula pennsylvanica stings. Autopsy revealed areas of demyelination throughout the central and peripheral nervous system with necrosis and inflammatory infiltration in the brain stem and spinal cord. Also, massive pulmonary embolism was found as a cause of death. Jin et al. (2010) reported that hyaluronidase is a minor yellow jacket venom allergen and only aboutb10% to 15% of patients with yellow jacket allergy was estimated to possess IgE against the hyaluronidase protein 131 . Component-resolved diagnosis with antigen and phospholipase detect all patients to have yellow jacket venom allergy. Witharana et al, (2015) in Sri Lanka conducted experiments on patients stings from 2011 to 2013. Data were gathered via a questionnaire conducted and specimens of offending insects were collected for identification. They found that five species were available from those in anaphylactic shock (four Apis dorsata, one Ropalidia marginata). Vespa tropica stinging leads to a characteristic skin lesion. They reported that the risk factors that favor the occurrence of disorders may include day-time outdoor activities, occupation (tea plantation workers) and period of year. The period of year highlights pollen season when the insects were found in the abundance. Only 4.6% of patients developed anaphylactic shock. Vespa tropica stings also lead to a unique skin lesion at the site of the sting.

### Scorpion sting

Scorpion stings were supposed to consider as a major threat for public health in many regions of the world especially in lessdeveloped countries of tropics and subtropics. Fatani et al. (2010) reported that scorpion was a real problem as compared to all other stings of insect in Saudi and Egyptian anti-venoms 132. The scorpion venomous species cause severe systemic reactions, lymphadenitis, twitching, muscle spasm and convulsions. The patients might die of respiratory paralysis with pulmonary edema within 2 to 3 hours after being sting 133. Fetaih et al. (2013) injected experimental mice with scorpion venom 134. They reported most obvious changes in the liver with acute cellular swelling, hydropic degeneration, congestion of central veins and portal blood vessels. Additionally, extramedullary hematopoiesis and invaginations in nuclei of hepatic cells with formation of intranuclear cytoplasmic inclusions were also observed 135. Local symptoms of envenomation by scorpion sting start within seconds or minutes after inoculation at the affected site. The systemic symptoms develop within 45 to 60 minutes after the sting and include Central Nervous System manifestations like irritability, anxiety, hyperthermia, excitability, agitation, visual changes, nausea, vomiting, nystagmus, hyperreflexia, ataxia, hemiplegia, focal or generalized seizures and encephalopathy 136. The scorpion toxin poorly passes through the blood-brain barrier and the effects on the Central Nervous System were secondary to the direct stimulation on the medullary sympathetic center 137. There was a sudden rise in blood pressure due to sympathetic stimulation, which can cause rupture of blood vessels, intracranial hemorrhage, encephalic infarcts, failure of the respiratory center and even paralysis 137.

### Other insects

Many reports are suggesting that mayflies may cause allergic rhinitis and asthma 32. Figley (1929) was the first to report the incidence of may fly allergy 1[32]. He reported that of the 1248 atopic patients studied 7% gave skin test positivity with Mayfly extract and that 40 of these patients benefited with immunotherapy. Pellicle was identified as body component of the mayfly responsible for the allergic symptoms. Parlato (1929) reported the first case of respiratory allergy to caddis fly 33. Skin tests with WBE of caddis fly resulted in immediate wheal and flare reaction in 5-7% of allergic patients. Hyposensitization (immunotherapy) of these patients with caddis fly extract resulted in good control of symptoms, particularly asthma 138-141. Osgood (1957) showed that 34.5% of his allergic patients showed marked reactivity to caddis fly WBE, while 60% elicited moderate and 5.5% slight to negative reactions 142. Kino and coworkers (1987) found that wings of caddis fly cause sensitization in asthmatic patients. Koshte and coworkers (1989) identified hemoglobin to be a prominent caddis fly allergen 143 . Smith and coworker (2005) described the prevalence of sensitization to commonly found insects like caddis fly 144. There were several reports on inhalant allergy to various other insects including housefly, mushroom fly, screwworm, blowfly and fruitfly, aphids, and bugs (Hemiptera), honey bees and yellow jacket (Hymenoptera), beetles (Coleoptera), locusts and crickets (Orthoptera) 138-146. Locust feces have been reported to be most potent allergen and were, therefore, been used for hyposensitization of allergic patients. Further, using immunefluorescent staining several workers suggested that the source of locust antigen might be the peritrophic membrane that lines the gut and surrounds the feces 147.

## Conclusion

It is quite evident that insects contribute clinically important inhalant allergens to the air in respirable sized particles. Therefore, it is of interest to review information on allergens caused by insects. However, the study on allergy caused by insects is limited. We are surrounded by a large number of other insect species and it is a potential source of inhalant allergens. Hence, we document known allergens caused by insects in this review.

## Figures and Tables

**Table 1:** Insect allergies prevalent across the major countries (Adapted from Anamika and Shruti Dutt, 2017).

Country	Cause of insect allergy
India	Bee, Yellow jackets, hornets, wasps
USA	Paper Wasp, Yellow Jacket, Hornet, and European Hornet
UK	Wasps and Hornets
Japan	The Killer Hornet-Suzumebachi, Mukade-Centipede, Huntsman spider, cockroaches
Australia	Lxodes, Australian Jack Jumper Ant
Africa	Bumblebee, Humblebee, Fire ant, Harvester ant

**Table 2:** Mueller grading system for systemic reaction to insect sting (Adapted from Tarun Kumar Dutta et al. 2013)

Grade type	Description
Grade 1	Systemic reaction is characterized by generalized urticaria or erythema, itching, malaise or anxiety
Grade 2	Reactions may include symptoms associated with grade I reactions as well as generalized edema, tightness in the chest, wheezing, abdominal pain, nausea and vomiting and dizziness
Grade 3	Reactions may include symptoms associated with grade I or II reactions of dyspnea, dysarthria, hoarseness, weakness, confusion and a feeling of impending down
Grade 4	Reactions may include symptoms associated with grade I, II or III reaction as well as any two of the following with fall in BP, loss of consciousness, incontinence of urine or feces or cyanosis

**Table 3:** Simon Brown three-tier system of classification.

Grade		Defined By
Mild	Skin and subcutaneous tissues only	Generalized erythema, urticaria, periorbital edema or angioedema
Moderate	Featured suggesting respiratory, cardiovascular or gastrointestinal involvement	Dyspnea, stridor, wheeze, nausea, vomiting, dizziness (presyncope), diaphoresis, chest or throat tightness or abdominal pain
Severe	Hypoxia, hypotension or neurologic compromise	Cyanosis or SpO2 less than 92 percent at any stage, hypotension (SBP less than 90 mm Hg in adults), confusion, collapse, LOC or incontinence

**Table 4:** Reactions due to caterpillars and moths

Type of reaction	Clinical features
Localized stinging reaction	The majority were caused by caterpillars.
	Cause varying degrees of pain, itchiness, weal or blister formation and rarely systemic symptoms such as dizziness, sweating, and abdominal pain.
Papular, urticaria and dermatitis	Usually caused by hairs from caterpillars or moths.
	No Lepidoptera species in NZ cause this type of reaction.
	Reactions range from mildly itchy, papular urticaria (small red bumps swelling) that resolves within an hour to moderately itchy, urticarial, scaly, blistering or widespread eczema-like reactions that can persist for weeks.
Widespread	Some species of Lonomia caterpillars found in South America cause localized stings that may progress to a severe haemorrhagic illness.
Hemorrhage (bleeding)	The sting transmits venom which causes burning pain, headache, nausea or vomiting.Over the next few days widespread bleeding occurs into the skin, mucous membranes, lungs, brain.
Ophthalmia nodosa	This is a toxic or allergic eye irritation caused by caterpillar hairs.
	The hairs may be windblown, transferred to the eye with a finger or other object or the caterpillar may contact the eye directly.
	Upper eyelid contact dermatitis usually occurs. Immediately after exposure, chemosis (swelling of the conjunctiva) develops.
Dendrolimiasis and pararamose	These refer to itchy skin rashes associated with joint pain or inflammation. Cartilage may also be involved in dendrolimiasis.
	Joint destruction and potentially deforming arthritis can results
